# Effect of a CGMS and SMBG on Maternal and Neonatal Outcomes in Gestational Diabetes Mellitus: a Randomized Controlled Trial

**DOI:** 10.1038/srep19920

**Published:** 2016-01-27

**Authors:** Qiong WEI, Zilin SUN, Yue YANG, Hong YU, Hongjuan DING, Shaohua WANG

**Affiliations:** 1 Department of Endocrinology, The Affiliated ZhongDa Hospital of Southeast University, No.87 DingJiaQiao Road, Nanjing, 210009, PR China; 2 Department of Obstetrics & Gynecology, The Affiliated ZhongDa Hospital of Southeast University, Nanjing, PR China; 3 Department of Obstetrics & Gynecology, Nanjing Maternity and Child Health Care Hospital, Nanjing, PR China

## Abstract

In this study, we sought to investigate the effects of a continuous glucose monitoring system (CGMS) on maternal and neonatal outcomes. A total of 106 women with gestational diabetes mellitus (GDM) in gestational weeks 24–28 were randomly allocated to the antenatal care plus CGMS group or the self-monitoring blood glucose (SMBG) group. The CGMS group was subdivided into early and late subgroups. There were no significant differences in prenatal or obstetric outcomes, e.g., caesarean delivery rate, Apgar score at 5 min, macrosomia or neonatal hypoglycaemia, between the CGMS and SMBG groups. The CGMS group had lower glycated haemoglobin (HbA1C) levels than the SMBG group; however, the difference was not statistically significant. The proportion of GDM women with excessive gestational weight gain was lower in the CGMS group than in the SMBG group (33.3% vs. 56.4%, *P* = 0.039), and women who initiated CGMS earlier gained less weight (*P* = 0.017). The mode of blood glucose monitoring (adjusted OR 2.40; 95% CI 1.030–5.588; *P* = 0.042) and pre-pregnancy BMI (adjusted OR 0.578; 95% CI 0.419–0.798; *P* = 0.001) were independent factors for weight gain. In conclusion, early CGMS for GDM mothers reduces gestational weight gain. A follow-up study with a large cohort is needed.

Gestational diabetes mellitus (GDM) is characterized by carbohydrate intolerance with an onset or first recognition during pregnancy^1^. Even mild hyperglycaemia in late gestation is associated with an elevated risk of complications for both the mother and her foetus^2^. In non-diabetic pregnant women with an abnormal screening result, mid-pregnancy HbA1c levels may predict neonatal birth weight, and they are related to the amniotic fluid index at 32–34 weeks of gestation^3^. Evidence has shown that early screening for and treatment of GDM reduce perinatal morbidity and improve post-delivery outcomes^4^. The most important factor in GDM management is glycaemic control to reduce adverse outcomes in pregnant women with GDM^5^,^6^. Blood glucose levels have become the “key player” for monitoring and directing treatment during pregnancy^7^. Excessive gestational weight gain (GWG) also increases the risk for adverse conditions during gestation^8^. The consequences of excessive weight gain include large-for-gestational age (LGA) neonates^9^, neonatal macrosomia, preterm labour, and caesarean delivery^10^. GDM and excessive GWG significantly affect the mother in later life; these conditions highly correlate with maternal obesity^11^,^12^. In addition to optimizing glycaemic control, these findings emphasize the need to control weight gain before and during gestation.

Glucose monitoring during pregnancy is indispensable for improving glycaemic control and reducing the risk of related adverse perinatal outcomes^13^. Self-monitoring of blood glucose (SMBG) is recommended for women with pre-gestational and gestational diabetes^14^,^15^. SMBG can effectively reduce the rate of foetal overgrowth in women with mild gestational diabetes^16^. A continuous glucose monitoring system (CGMS) effectively detects postprandial glucose peaks^17^, hyperglycaemia, and hypoglycaemia^18^. Several randomized and controlled clinical trials have shown that CGMS is superior to traditional frequent blood glucose monitoring in terms of enhanced metabolic control, an ideal infant birth weight, and a reduced risk of macrosomia^19^,^20^,^21^,^22^,^23^. In a recent randomized trial, the intermittent use of real-time CGM during pregnancy, in addition to self-monitoring plasma glucose seven times daily, did not improve glycaemic control or pregnancy outcomes in women with pre-gestational diabetes^24^.

Prospective randomized clinical trials are warranted to confirm the effects of a CGMS, especially when initiated early, on glycaemic control as well as maternal and neonatal outcomes, including prenatal and obstetrical outcomes, among GDM mothers; this represents the purpose of this study, and we sought to determine whether CGMS reduces GWG. To the best of our knowledge, no study has examined the effects of behavioural adjustments, such as attentive and early glycaemic monitoring, on the GWG of GDM mothers.

## Methods

### Ethics

The protocol and informed consent documents were approved by the research ethics committees of ZhongDa Hospital, which is affiliated with Southeast University. This study was performed in accordance with approved guidelines. All patients provided written informed consent prior to participation in the study protocol.

### Study design and patient population

This study was a prospective, observational, open-label randomized controlled trial undertaken in the Department of Endocrinology, Zhongda Hospital, the Affiliated Hospital of Southeast University, China, from September 2011 to December 2012.

The mothers enrolled in this study were diagnosed after 24 weeks of gestation. The model of care included lifestyle advice, clinical follow-up, and glucose monitoring combined with CGMS or SMBG. The CGMS group was subdivided into early and late subgroups. The patients in the two subgroups were asked to wear a CGMS during their second and third trimesters of pregnancy.

**Figure 1:**
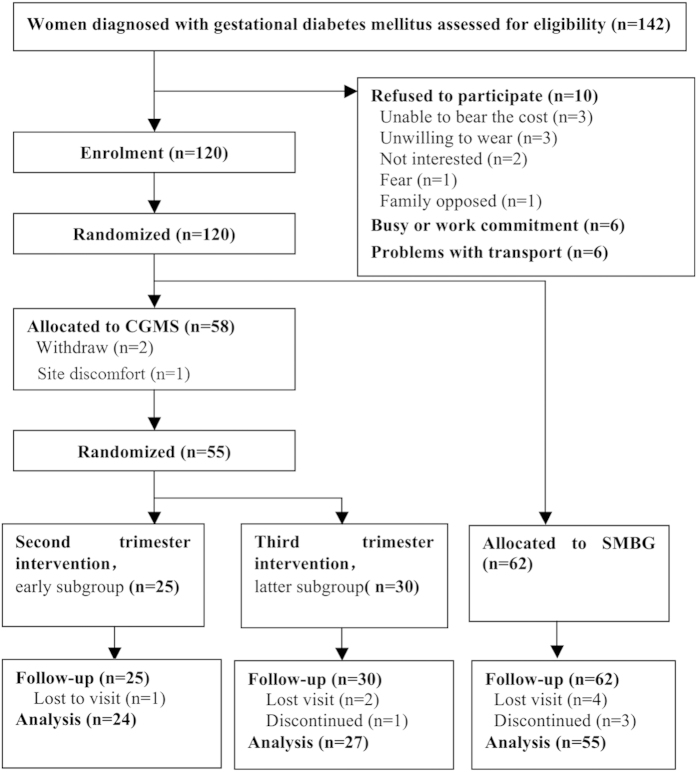
Patients’ progress throughout the study.

All the enrolled women underwent a 75 g oral glucose tolerance test (OGTT) at 24 to 28 weeks of gestation according to the criteria of the American Diabetes Association (ADA)^25^. Based on the one-step approach recommended by the World Health Organization^26^, the International Association of Diabetes in Pregnancy Study Groups^27^, and the ADA^25^, the pregnant women were defined as having GDM if they had at least one abnormally high plasma glucose value out of the three measurements in the 75 g OGTT (fasting >92 mg/dL (5.1 mmol/L), 1 h > 180 mg/dL (10.0 mmol/L), or 2 h > 153 mg/dL (8.5 mmol/L)). The inclusion criteria were as follows: between 24 and 28 weeks gestation with a singleton pregnancy, a positive oral glucose challenge result, and written informed consent. The exclusion criteria were as follows: a diagnosis of diabetes mellitus, previous treatment for GDM, presence of infection, or other severe metabolic, endocrine, medical or psychological comorbidities. The patients were randomly allocated to either the antenatal care plus CGMS group or the SMBG group after GDM diagnosis by a computer generated random number table. The patients in the CGMS group were randomly allocated in a similar manner to the early and late subgroup. The patients in the early and late subgroups were asked to wear a CGMS during gestational weeks 24 to 28 (second trimester) or 28 to 36 (third trimester), respectively (Fig. 1).

All the women were interviewed in the hospital and underwent dietary counselling for a eucaloric diet with a low glycaemic index and low saturated fat levels. A basic explanation of the nutrient composition of commonly consumed food and food products was provided. The dietary advice dictated that 50% to 60% of the energy per day should be derived from carbohydrates, 25% to 30% should be from fat, and 15% to 20% should be from protein; furthermore, energy intake should be distributed as equally as possible throughout the day, based on the recommendations of the China Diabetic Association. Total calories were approximately distributed as 10% for breakfast, 30% each for lunch and dinner, and 30% for snacks. The diet was divided into three meals and three snacks. During pregnancy, moderate intensity physical exercise was encouraged. The heart rate goals for moderate intensity exercise were evaluated by the formula (220-age) × 0.65–0.75. The physical conditioning programme was a 35- to 45-min session performed thrice weekly. The physiotherapist motivated the women individually to continue exercising during pregnancy or to start exercising. The physiotherapist also provided written instructions for exercise and self-care. Walking, swimming, and cycling are recommended types of exercise^28^. Ketonuria was monitored during pregnancy every month to avoid starvation ketosis. The evaluation parameters included age, pre-pregnancy BMI, current weight gain during pregnancy, family history of diabetes, hypertension in pregnancy, glycated haemoglobin (HbA1C) levels at diagnosis, glucose levels determined by the diagnostic OGTT, fasting plasma insulin, fasting C-peptide, postprandial 2 h C-peptide, high-density lipoprotein (HDL) cholesterol, and triglycerides. The homeostasis model assessment of insulin resistance (HOMA-IR) was used to assess insulin resistance: [fasting glucose (mmol/L) × fasting insulin (mU/L)]/22.5^29^,^30^.

The treatment programme was determined based on self-monitored plasma glucose values within a week after the initiation of monitoring. Insulin treatment was administered under conditions of two fasting blood glucose values >105 mg/dL (5.8 mmol/L), two 1 h postprandial blood glucose levels >155 mg/dL (8.6 mmol/L), 2 h postprandial blood glucose >130 mg/dL (7.2 mmol/L), or fasting blood glucose >90 mg/dL (5.5 mmol/L) with at least two postprandial values >141 mg/dL (7.8 mmol/L), according to the ADA. Women received treatment with NPH as an intermediate-acting insulin with an initial dose of 0.2 units/kg. If fasting blood glucose levels were high, then treatment was given before bedtime. If postprandial blood glucose levels were high, then regular insulin or short-acting insulin was administered before meals (1 unit for every 101 umg/dL (0.561 ummol/L) over the target value)^31^.

Follow-up meetings were set every 2 to 4 weeks until 28 gestational weeks, fortnightly until 32 gestational weeks and weekly thereafter. The side effects of pregnancy were documented at the prenatal visits. All the patients underwent an ultrasound evaluation of foetal weight in the third trimester. The patients with dietary counselling had a routine check at 36 gestational weeks.

### SMBG and CGMS in subjects

The patients in the SMBG and CGMS groups were taught to perform self-monitoring of blood glucose by using Accu-Chek Advantage metres (Roche Diagnostics, Mannheim, Germany). These patients were instructed to check their glucose level four times a day (fasting and 1 hour after the beginning of each meal) from the first visit at which they received the GDM diagnosis until labour and delivery, except during the period mentioned below for the CGMS group. The CGMS group was monitored using a CGMS (Gold Medtronic MiniMed, Northridge, CA, USA). The CGMS sensor was inserted into the upper outer buttock of the subjects for 48 to 721 uhours on weekdays^32^. While subjects wore the CGMS sensor, glucose levels (at bedtime and 1 hour before the beginning of each meal) were monitored using Accu-Chek Advantage metres (Roche Diagnostics, Mannheim, Germany) and were input into the CGMS instrument as a calibration four times a day.

### Obstetrical and neonatal outcomes

The obstetrical and neonatal outcomes included caesarean section, birth weight, standard deviation of weight for gestational weeks, and Apgar score at 5 min. Neonatal hypoglycaemia was defined as a blood glucose concentration ≤45 mg/dL (2.5 mmol/L)^33^. Blood glucose concentrations were measured in capillary blood samples that were obtained by using a heel-prick lance. Macrosomia was characterized as a mean birth weight >4000 g. Large-for-gestational age (LGA) was defined as birth weight above the 90^th^ percentile, extreme LGA was defined as birth weight at or above the 97.7^th^ percentile, and small-for-gestational age (SGA) was defined as birth weight at or below the 10^th^ percentile based on standard growth and development tables for the Chinese population.

### Maternal glycaemic control

HbA1C levels were examined once every 4 weeks. Glycaemic control was also assessed via participant-monitored blood glucose levels, which were recorded four times a day. The measures were evaluated by comparing the CGMS data, including hypoglycaemia, which was defined as <59 mg/dL (3.3 mmol/L) during pregnancy; standard deviation (SD) of the mean glucose value; mean of daily continuous 24 h blood glucose (MBG); and mean amplitude of glucose excursions (MAGE) computed as the arithmetic mean of the differences between consecutive peaks and nadirs. Only increases of more than one SDBG of the mean glycaemic values were considered. The mean postprandial glucose excursion (PPGE) was measured. The absolute mean of the daily differences (MODD) was the mean absolute value of the differences between glucose values during two successive 24 h periods^34^.

### Pre-pregnancy BMI and gestational weight gain

Each woman reported her pre-pregnancy weight, and BMI was calculated as weight in kilograms divided by the square of height in metres. Self-reported pre-pregnancy weight is widely used in studies^35^,^36^ because most women do not have a preconception visit during which weight is measured. Pre-pregnancy BMI was classified as normal weight (BMI < 25 kg/m^2^), overweight (25 ≤ BMI < 30 kg/m^2^), or obese (BMI ≥ 30 kg/m^2^). GWG was calculated as the simple difference between the weight at the end of pregnancy and the pre-pregnancy weight. We measured the weight on the delivery day using the same medical scales (model 704, Seca, Hamburg, Germany) while the women were wearing light indoor clothes and no shoes. The 2009 Institute of Medicine (IOM) guidelines were used to classify the total gestational weight gain based on the pre-pregnancy BMI as follows: normal, 11.5 kg to 16 kg; overweight, 7 kg to 11.5 kg; and obese, 5 kg to 9 kg^37^. Differences in the proportions of women in each category of gestational weight gain based on the IOM guidelines were compared. Women were classified as having excessive gestational weight gain if they exceeded such standards.

### Statistical analysis

Quantitative and nominal data are presented as the mean ± SD and percentages, respectively. For the statistical analyses, Fisher’s exact test, Pearson’s *χ*^*2*^ test, and Student’s *t*-test were used when appropriate. Continuous variables were analysed using a two tailed t-test, and a *P* value less than <0.05 was considered statistically significant. For the prospective study, binary logistic regression analysis was performed to detect independent predictors of weight gain. All the statistical analyses were performed using statistical software (SPSS for Windows, version 11.5).

## Results

### Patient characteristics

Of the women who were preliminarily eligible to participate in the study, 55 in the CGMS group and 62 in the SMBG group provided informed consent to participate. However, 4 of the participants in the CGMS group were lost to follow-up and discontinued the study, and 7 of the participants in the SMBG were found to be ineligible. The final analyses included 51 participants in the CGMS group (24 wore the CGMS during the second trimester, and 27 wore the device in the third trimester) and 55 in the SMBG group. Of the randomized patients, 106 were included in the analysis (Fig. 1). The clinical features of the women in the SMBG and CGMS groups are listed in Table 1. They did not differ in age, education, family history of diabetes, pre-pregnancy BMI, hypertension or clinical data. Clinical data included the 2 h OGTT results at any individual time point (0 h to 2 h), C-peptide levels, and the HOMA-IR value. Data above the baseline were not significantly different between the early and late CGMS subgroups.

### Obstetrical and neonatal outcomes

The caesarean delivery rate was greater in the SMBG group than in the CGMS group, but the difference was not statistically significant (69% vs. 60%, *P* = 0.37). No births occurred before the 35^th^ gestational week. No perinatal deaths were observed in either group. Gestational weeks at delivery, Apgar score at 5 min, macrosomia, neonatal hypoglycaemia, and extreme LGA (≥97.7^th^ percentile) and SGA (≤10^th^ percentile) were not significantly different between the two groups. Fewer LGA (≥90^th^ percentile) infants were born to mothers in the CGMS group than to those in the SMBG group, but the difference was not statistically significant (35.3% vs. 52.7%, *P* = 0.071).

### Maternal glycaemic control

**Figure 2:**
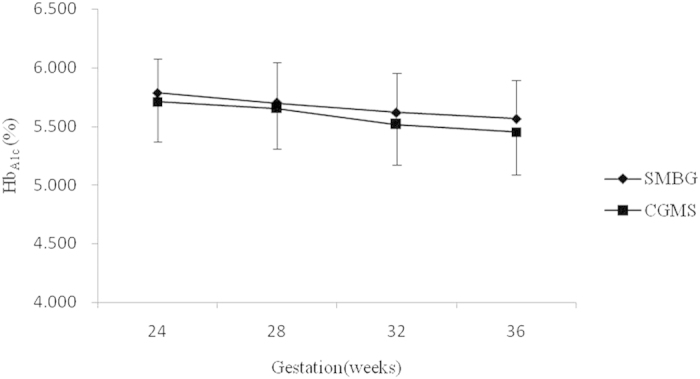
Mean HbA1C levels every four weeks in the CGMS and SMBG groups. Vertical lines represent the standard deviation at each time point.

HbA1C levels dropped slowly during the gestation period from baseline in both the CGMS and SMBG groups (5.7% ± 0.34% vs. 5.8% ± 0.29%, *P* = 0.096). Compared to those in the SMBG group, HbA1C levels were lower in the CGMS group but were not significantly different throughout the last two trimesters. Similar reductions in HbA1C levels were observed in the CGMS and SMBG groups (5.5% ± 0.39% vs. 5.6% ± 0.35%, *P* = 0.089) in later pregnancy (32 to 36 weeks gestation) (Fig. 2).

The continuous glucose monitor was commonly well tolerated by the pregnant women in the CGMS group. No skin infections occurred at the sensor insertion site, but mild erythema, itchiness, and inflammation often occurred. An average of 568 ± 30 glucose measurements were recorded, and the reported hypoglycaemic episodes occurred primarily during early morning and early evening. No significant differences in the mean and standard deviation of the glucose value, breakfast PPGE (BPPGE), lunch PPGE (LPPGE), dinner PPGE (DPPGE), and MODD were observed between the subgroups. As expected, MAGE was significantly higher among mothers with the CGMS in the third trimester than among those wearing the CGMS in the second trimester (4.21 ± 0.45 vs. 4.01 ± 0.14, *P* = 0.046) (Table 2).

Insulin was more commonly used in the CGMS group than in the SMBG group (31.3% (16 patients) vs. 12.7% (7 patients), *P* = 0.02). Among the 16 patients in the CGMS group who used insulin, 11 used regular insulin alone as a short-acting insulin 1–3 times per day. Five patients used intermediate-acting insulin; 1 of these patients was treated with intermediate-acting insulin alone, and 4 were treated with intermediate-acting insulin and regular insulin. Among the 7 patients in the SMBG group who used insulin, one patient each used regular insulin alone and NPH alone; the remaining 5 patients were treated with NPH and regular insulin.

In a comparison of the two groups, regular insulin was more commonly used in the CGMS group (68.8% vs. 14.3%, *P* = 0.027), and NPH was more commonly used in the SMBG group (71.4% vs. 25.0%, *P* = 0.066).

By the last visit, there was no significant difference in the required insulin dose between the CGMS and SMBG groups (34.23 IU ± 10.39 IU vs. 30.85 IU ± 8.87 IU; *P* = 0.45).

### Pre-pregnancy BMI and gestational weight gain

No significant difference in pre-pregnancy BMI was observed between the CGMS and SMBG groups (Table 2). The GDM women in the CGMS group gained significantly less weight than those in the SMBG group (13.56 kg ± 2.81 kg vs. 14.75 kg ± 2.91 kg, *P* = 0.004). Compared with the SMBG group, the CGMS group had a lower proportion of patients who experienced excessive gestational weight gain (33.3% vs. 56.4%) and a higher proportion of patients with appropriate weight gain (62.8% vs. 38.2%). Fewer patients wearing the CGMS gained an inadequate amount of gestational weight (3.9% vs. 5.5%, *P* = 0.039). Furthermore, the GDM women who wore the CGMS in the early stage gained less weight than those who wore the CGMS in the later stage (12.72 kg ± 2.83 kg vs. 14.31 kg ± 2.64 kg, *P* = 0.003) (Table 3).

Binary logistic stepwise regression analysis was performed to assess the independent effects of clinical factors on weight gain for GDM mothers. The following factors were considered: age, family history of diabetes, education, pre-pregnancy BMI, OGTT 0 h, OGTT 1 h, OGTT 2 h, fasting C-peptide, postprandial C-peptide, SBP, DBP, TG, LDL-C, HDL-C, HbA1C, insulin medication, and mode of blood glucose monitoring. Pre-pregnancy BMI and mode of blood glucose monitoring entered the model, whereas the other independent variables failed to enter. The mode of blood glucose monitoring (adjusted OR 2.4; 95% CI 1.030–5.588; *P* = 0.042) and pre-pregnancy BMI (adjusted OR 0.578; 95% CI 0.419–0.798; *P* = 0.001) were independent factors for weight gain.

## Discussion

The CGMS provides a detailed depiction of glycaemic variability. However, whether the generated readings improve pregnancy outcomes is unclear. Our results suggest that the use of a supplementary CGMS by GDM mothers was associated with reduced weight gain. In addition, lower maternal weight gain was observed when the CGMS was used during the second trimester rather than during the third trimester. This result implies that early implementation of the CGMS can reduce weight gain among women with GDM. No significant differences in other prenatal or obstetrical outcomes were observed between the CGMS and SMBG groups. The CGMS group had a lower proportion of LGA neonates and lower HbA1C levels than the SMBG group; however, these differences were not statistically significant.

The 2009 IOM guidelines recommend narrow and specific GWG ranges that are evaluated on the basis of pre-pregnancy BMI. Fewer women had excessive GWG in the CGMS group than in the SMBG group. However, Kestilä *et al.*^38^ have reported that CGMS is not superior to SMBG in maintaining normal weight among GDM mothers. Notably, their study included only a few high-risk women and did not adopt the 2009 IOM guidelines for distinguishing patient weight. In the current study, more mothers in the CGMS group used insulin; several possible explanations may have contributed to their ability to maintain their weight. SMBG is generally conducted; more than 90% of obstetricians recommend that GDM patients measure fasting blood glucose (FBG)^39^, and only 61% of obstetricians recommend that these patients undergo 2-hour postprandial tests^40^. However, the main goal of dietary adjustments is to eliminate peaks in blood glucose levels; the 1–2 h postprandial period is the recommended period for testing blood glucose levels in women with GDM. A CGMS can measure postprandial glucose peaks in an uninterrupted manner that is more efficient than SMBG; thus, a patient’s dietary plan can be adjusted in accordance with the CGMS results. As a result, excessive caloric intake is avoided. Furthermore, a CGMS improves the glycaemic profiles of pregnant women with insulin-treated diabetes. Although no significant difference emerged, maternal HbA1C levels, which reflect mean blood glucose levels over the preceding 4–6 weeks, began to fall after the 28th gestational week in the CGMS group. Improved glycaemic control is probably associated with a lower birth weight and a reduced risk of macrosomia. Among pregnant women with diabetes, continuous glucose monitoring during pregnancy is associated with improved glycaemic control in the third trimester, lower birth weight, and a reduced risk of macrosomia^18^. Additionally, continuous glucose monitoring data are used as an educational tool to optimize lifestyle and therapeutic management, which evokes gradual improvements in glycaemic profiles during pregnancy. Combining a healthy lifestyle with CGMS intervention after a GDM diagnosis may increase awareness among patients of adverse obstetrical and neonatal outcomes, reinforce gradual improvements in glycaemic profiles throughout pregnancy, and offset weight gain due to the use of insulin.

The subgroup analysis showed that GDM mothers who used the CGMS during early gestation gained less weight than those who used the CGMS during late gestation. The second trimester is the most sensitive period of maternal weight gain^41^. According to guidelines, excessive GWG can be predicted among overweight and obese mothers during this period^42^. In this study, gestational weeks 24–28 were defined as the second trimester subgroup. Our data showed that using the CGMS during the second trimester prevented excessive GWG. Several indices of glycaemic variability (MAGE, SD, BPPGE, LPPGE, DPPGE, and MODD) were also evaluated. These indices, particularly MAGE, increased among GDM patients during the third trimester. These increases contributed to excessive weight gain.

Multivariate conditional regression analysis showed that pre-pregnancy BMI was an independent predictor of maternal weight gain. LGA new-borns and insulin administration are prevalent among overweight mothers^43^. Women with a high pre-pregnancy BMI are susceptible to increased weight gain during gestation. Thus, pre-pregnancy obesity (overweight) should be controlled during the pre-gestation period^44^.

No significant differences in adverse perinatal outcomes or glycaemic control were observed between the CGMS and SMBG groups or between the early and late CGMS subgroups. These findings can be attributed to the small sample size. A previous study with a similarly small sample size also failed to detect significant differences in maternal/foetal outcome variables, such as HbA1C, gestational age at delivery, infant birth weight, and neonatal hypoglycaemia, between the CGMS and SMBG groups^38^,^45^. However, Murphy *et al.*^23^ have shown that the CGMS improves glycaemic control in the third trimester, lowers birth weight, and reduces the risk of macrosomia among diabetic pregnant women. These differences might be attributed to the study population and setting. The present study confirmed the previous finding that CGMS increases the use of antihyperglycaemic drug therapy^30^; insulin use was more common in the CGMS group than in the SMBG group. Our results also showed that regular insulin was more commonly used in the CGMS group and that NPH was more commonly used in the SMBG group. By the subjects’ last visit, there was no significant difference in the required insulin dose between the two groups. These data demonstrated the advantages of the CGMS in the accurate detection of hyper- and hypoglycaemia, e.g., undetected nocturnal hypoglycaemia and postprandial hyperglycaemia. Therefore, by use of CGMS, the use of regular insulin could be increased, and the use of NPH could be decreased before bedtime. This finding might explain why some GDM patients satisfied the criteria for insulin treatment. The CGMS data help clinicians detect more blood glucose excursions than a finger-prick diary record, and the values reflect the more reasonable therapy and better glucose control. CGMS is an effective tool for facilitating therapeutic changes when necessary.

The interpretation of the data presented in this study has some limitations. First, the education management was not blinded; thus, the Hawthorne effect cannot be excluded. Patients who were counselled on weight gain, nutrition, and exercise likely considered these factors and modified their lifestyle. Second, the small cohort of recruited patients and the few perinatal complications possibly limited the generation of statistically significant results. The same reason deterred us from constructing a model that might elucidate the role of the relevant variables. Third, some clinical data (e.g., sensor data on instrument failure, instrument error, pain, and discomfort) were unavailable. Follow-up data at 6 weeks postpartum were also deficient, and the incidence of diabetes among the mothers was not examined.

Despite these limitations, this study proved that the CGMS, especially when initiated early, provides benefits in conjunction with a professional healthcare system to reduce maternal weight gain and glycaemic variability. Extensive clinical studies are warranted to test the effectiveness of CGMS management of maternal weight gain in reducing perinatal problems, especially foetal macrosomia, in GDM women.

## Additional Information

**How to cite this article**: WEI, Q. *et al.* Effect of a CGMS and SMBG on Maternal and Neonatal Outcomes in Gestational Diabetes Mellitus: a Randomized Controlled Trial. *Sci. Rep.* **6**, 19920; doi: 10.1038/srep19920 (2016).

## Figures and Tables

**Table 1:** Maternal characteristics in women with gestational diabetes mellitus allocated to CGMS or SMBG.

	SMBG	CGMS	*P*-value	CGMS-early	CGMS-latter	*P*- value
Number	55	51	—	24	27	—
Maternal age (years)	29.96 ± 3.43	30.29 ± 3.60	0.63	30.20 ± 3.64	30.37 ± 3.63	0.875
Education beyond high school (n[%])	32(58.1)	28(54.9)	0.734	16(66.7)	12(44.4)	0.111
Family history of diabetes (n[%])	7(12.7)	9(17.6)	0.480	4(16.7)	5(18.5)	1.000
Pre-pregnancy BMI			0.824			0.105
normal weight(<25) (n[%])	40(72.7)	39(76.5)		21(87.5)	18(66.7)	
overweight or obese (BMI ≥25) (n[%])	15(27.3)	12(23.5)		3(12.5)	9(33.3)	
OGTT 0 h (mmol/L)	5.67 ± 0.29	5.69 ± 0.58	0.859	5.67 ± 0.55	5.71 ± 0.63	0.833
OGTT1h (mmol/L)	10.90 ± 0.85	10.86 ± 1.01	0.843	10.77 ± 1.03	10.94 ± 1.02	0.553
OGTT2h (mmol/L)	8.29 ± 0.94	8.23 ± 1.78	0.833	8.44 ± 2.38	8.05 ± 1.02	0.429
HbA1C at OGTT (%)	5.8 ± 0.29	5.7 ± 0.34	0.096	5.7 ± 0.30	5.6 ± 0.38	0.892
Fasting C-peptide(ng/ml)	2.34 ± 0.50	2.18 ± 0.45	0.083	2.24 ± 0.51	2.12 ± 0.40	0.367
C-peptide 2 h(ng/ml)	7.35 ± 1.67	7.01 ± 1.95	0.327	7.11 ± 2.22	6.93 ± 1.71	0.739
HOMA-IR	3.61 ± 1.16	4.01 ± 1.83	0.181	4.07 ± 1.57	3.95 ± 2.06	0.834
HDL-C(mmol/L)	1.66 ± 0.37	1.73 ± 0.34	0.288	1.78 ± 0.38	1.68 ± 0.29	0.299
LDL-C (mmol/L)	2.69 ± 0.70	2.91 ± 0.55	0.065	2.91 ± 0.48	2.92 ± .0.61	0.720
Triglycerides (mmol/L)	2.57 ± 0.74	2.86 ± 1.12	0.105	2.88 ± 1.32	2.85 ± 0.92	0.918
SBP (mm Hg)	119.9 ± 12.1	117.7 ± 9.4	0.287	115.7 ± 8.1	119.4 ± 10.2	0.163
DBP(mm Hg)	72.3 ± 7.8	71.1 ± 7.8	0.405	70.8 ± 7.5	71.3 ± 8.2	0.834
Mean arterial pressure(mm Hg)	88.2 ± 8.1	86.5 ± 8.4	0.276	85.4 ± 7.4	87.52 ± 8.2	0.329

Data are expressed as means ± SD or n (%) of patients. BMI, body mass index; OGTT, oral glucose tolerance test; HbA1C, glycated haemoglobin A1C; HOMA-IR, homeostasis model assessment index-insulin resistance; HDL-C, high-density lipoprotein cholesterol; LDL-C, low-density lipoprotein cholesterol; SBP,systolic blood pressure; DBP, diastolic blood pressure.

**Table 2:** Glucose excursion at the second and third trimester of pregnancy in women with gestational diabetes.

	CGMS-early	CGMS- latter	*P* -value
Number	24	27	—
MBG (mmol/L)	6.81 ± 0.62	6.98 ± 0.46	0.264
SD (mmol/L)	1.14 ± 0.24	1.10 ± 0.18	0.485
MAGE (mmol/L)	4.01 ± 0.14	4.21 ± 0.45	0.046*
BPPGE (mmol/L)	5.080 ± 0.90	5.43 ± 0.52	0.090
LPPGE (mmol/L)	2.71 ± 0.37	2.82 ± 0.55	0.420
DPPGE (mmol/L)	3.25 ± 0.44	3.42 ± 0.55	0.227
MODD (mmol/L)	1.10 ± 0.37	1.21 ± 0.28	0.263

Data are expressed as means ± SD of patients. MBG, mean of daily continuous 24 h blood glucose; SD, standard deviation of the mean glucose value; MAGE, mean amplitude of glucose excursions; BPPGE, mean postprandial glucose excursions of breakfast; LPPGE, mean postprandial glucose excursions of lunch; DPPGE, mean postprandial glucose excursions of dinner; MODD, mean absolute value of the differences between glucose values during two successive 24 h periods.

**Table 3:** Pregnancy outcome in women with gestational diabetes mellitus allocated to CGMS or SMBG.

	SMBG	CGMS	*P*-value	CGMS-early CGMS-latter	*P*- value	
Number	55	51	—	24	27	—
Caesarean section (n[%])	38(69)	31(60)	0.370	13(54.2)	18(66.7)	0.361
birth weight (g)	3451.09 ± 514.05	3275.88 ± 519.72	0.084	3192.50 ± 458.25	3350.88 ± 567.04	0.285
Apgar 5 min	9.49 ± 0.50	9.40 ± 0.56	0.39	9.41 ± 0.56	9.38 ± 0.56	0.861
Macrosomia, (n[%])	7(12.7)	4(7.8)	0.410	1(4.2)	3(11.1)	0.690
Large for gestational age(≥90^th^ centile)(n[%])	29(52.7)	18(35.3)	0.071	6(25.0)	12(44.4)	0.147
Extremely large for gestational age(≥97.7^th^ centile)(n[%])	17(30.9)	9(17.6)	0.113	3(12.5)	6(22.2)	0.588
Small for gestational age(≤10^th^ centile)(n[%])	2(3.6)	2(3.9)	1.000	0(0)	2(7.4)	1.000
Neonatal hypoglycemia(n[%])	7(12.7)	4(7.8)	0.410	0(0)	4(14.8)	1.000
Treated medically(n[%])	7(12.7)	16(31.3)	0.020*	7(29.1)	9(33.3)	0.749
Gestational weeks at birth	37.47 ± 1.32	37.44 ± 0.99	0.922	37.18 ± 0.98	37.66 ± 0.950	0.084
Gestational weight gain(n[%])			0.039*			0.017*
Excessive weight gain	31(56.4)	17(33.3)		4(16.7)	13(48.1)	
Inadequate gain	3(5.5)	2(3.9)		1(4.2)	1(3.2)	
Appropriate gain	21(38.2)	32(62.7)		19(79.1)	13(48.1)	

Data are expressed as means  ±  SD or n (%) of patients.
